# Disseminate Fungal Infection after Acute Pancreatitis in a Simultaneous Pancreas-Kidney Recipient

**DOI:** 10.1155/2010/898245

**Published:** 2010-06-07

**Authors:** Anna Rossetto, Umberto Baccarani, Dario Lorenzin, Andrea Risaliti, Pierluigi Viale, Vittorio Bresadola, Gian Luigi Adani

**Affiliations:** ^1^Department of Surgery & Transplantation, University Hospital of Udine, 33100 Udine, Italy; ^2^Department of Surgery & Transplantation, University Hospital of Ancona, 60100 Ancona, Italy; ^3^Department of Infectious Diseases, University Hospital of Udine, 33100 Udine, Italy

## Abstract

Fungal infections after kidney transplantation are a major cause of morbidity and mortality, and *Candida* infection of the pancreas is considered an infrequent but important agent in necrotizing pancreatitis. We report the case of a 43-year-old Caucasian patient who underwent simultaneous pancreas-kidney transplantation because of diabetes type I, and chronic renal failure with peritoneal dialysis. The postoperative course was complicated by acute pancreatitis due to the thrombosis of the splenic artery of the graft, the subsequent acute rupture of the external iliac artery caused by fungal arteritis (*Candida glabrata*), and peritonitis a few days later caused by sigmoid perforation with detection of *Candida glabrata* infection of the resected intestinal tract. The present case remarks that awareness and prevention of fungal infection are major issues in the transplant field. Important information can be added by systematic culture of conservation perfusates but, probably, the best way for early recognition of a critical level of infectious risk remains the routine application of the colonization index screening. In cases of positive results, preemptive antifungal therapy could be warranted.

## 1. Introduction

Infections remain a major challenge among transplant recipients. In the early posttransplant phase the most common [1] are local or systemic severe *Candida spp*. infections, either endogenous or exogenous [2]. 

A risk factor analysis has shown that patients with severe injury of the pancreas are significantly more prone to develop a *Candida* infection [3]. We describe a disseminated fungal infection as a complication of acute pancreatitis of the graft after simultaneous pancreas-kidney transplantation (SPKT).

## 2. Case Report

A 43-year-old Caucasian male suffering from type 1 diabetes and chronic renal failure with peritoneal dialysis underwent SPKT in December 2008. The donor was a 26-year-old male; the cross-match was negative. The transplant procedure was performed with no complications: the pancreas venous outflow was systemic via an end-to-side anastomosis between the portal vein of the graft and the recipient's vena cava. The inflow was restored through an end-to-side anastomosis between the donor's Y iliac graft and the recipient's common iliac axis. An enteric exocrine drainage was carried out on a jejunal loop. The kidney was transplanted intraperitoneally with arterial and venous anastomosis on the left external iliac axis. The cold and warm ischemia times were 355 and 510 minutes, 45 and 35 minutes for the pancreas and the kidney, respectively. 

**Figure 1:**
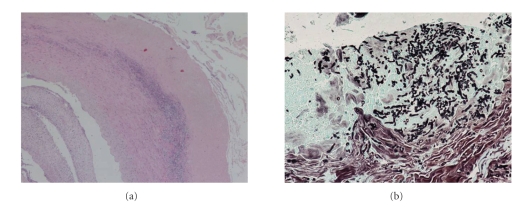
(a) 10x, Hematoxylin and eosin. Arterial wall with necrosis and inflammatory infiltrates; (b) 20x, Grocott. Fungal iphae in the arterial wall.

Antibiotic surgical prophylaxis with intravenous cefazolin (2 grams single dose) was administered. Immunosuppressive therapy consisted of Basiliximab, Tacrolimus, Steroids, and Mofetil Mycophenolic. Eight days after transplantation the pancreas had to be removed because of the development of acute pancreatitis due to the thrombosis of the splenic artery of the graft. Empirical antimicrobial therapy with Piperacillin-Tazobactam (2.2 grams X4/day i.v) and Fluconazole (400 mg/day i.v.) was administered. On postoperative day 7 the patient underwent emergency operation. A successful suture of the right external iliac artery was performed because of an acute rupture. Histological examination of the artery evidenced fungal arteritis by *Candida Glabrata* (*C. Glabrata*) with extended necrosis (Figures 1(a) and 1(b)), and culture yielded *C. Glabrata, *with dose-dependent fluconazole and itraconazole sensitivity (S-DD).

**Figure 2:**
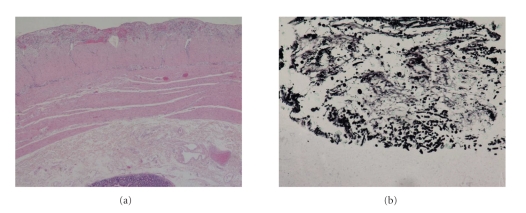
(a) 10x, Hematoxylin and eosin. Phlogosis of the intestinal sierosa; (b) 20x, Grocott. Fungal iphae in the arterial wall.

Antimycotic therapy with Caspofungin **(**50 mg/day after a loading dose of 70 mg**) **was immediately started. Again, eight days later, the patient developed peritonitis and underwent another emergency operation; a Hartmann procedure was performed on the intraoperative finding of single perforation of the sigmoid colon. Histological examination of the intestinal tract evidenced the presence of fungal spores and *C. Glabrata *grew from culture of surgical specimens (Figures 2(a) and 2(b)), although the patient had already started antimycotic therapy with Capsofungin (effective on *C. Glabrata*). The antimtycotic therapy, started 8 days before, was apparently insufficient to eradicate *C. Glabrata* completely. On histological examination the surgical specimen revealed, besides fungal iphae, ischemic type lesions, probably responsible for perforation.

The postoperative course was regular, the patient was switched from Caspofungin (total therapy 25 days) to Fluconazole (400 gm/die  administered orally), and was discharged from hospital in good general conditions. One year follow-up has been regular.

## 3. Discussion

*Candida* is more likely to cause infection in patient having surgery for acute pancreatitis than in those with either gastrointestinal perforations or other surgical conditions [4–6]. In the transplantation field, arterial infection associated with *Candida *is an emerging problem. Some authors suggest that the infection is transmitted from the donor, with a potential source of infection represented by the contaminated graft, whereas the variability of the infectious risk is related to the duration and complexity of the harvesting and storage process [7]. However, in our case no risk factors or positive fungal cultures were reported from the donor; no signs or symptoms suggestive of systemic fungal infection were present in our case before transplant. Blood and urinary cultures were always negative. The patient had a major risk factor for *Candida* infection, represented by a history of peritoneal dialysis [8], but no episodes of peritonitis or other infections had been reported before transplant. Moreover during the post transplant phase, no blood cultures were positive for *Candida spp*.

Therefore, while the colonic localization of *Candida spp* might be related to an endogenous source, the development of the arteritis is more difficult to explain without any episode of candidemia and graft contamination remains the more reasonable pathogenetic mechanism of infection [9]. 

From a therapeutic point of view, it is worth remarking that invasive Candidiasis developed despite Fluconazole therapy; although the isolation of an S-DD *C. Glabrata* strain well explains the Fluconazole failure [10].

In literature, the incidence of gastrointestinal complications in renal transplantation is relatively high, about 20%. Many predisposing factors have been suggested as steroids use, uremia and wound-healing capacity, chronic constipation, atherosclerotic change of the colon vascularization, and so forth, although the main causes reported are complication of a preexisting diverticular disease and intestinal ischemia [11, 12]. 

In conclusion, this case points out that awareness and prevention of fungal infections are major issues in the transplant field. The best way for the early recognition of infectious risk remains the routine application of the colonization index screening [13], followed by preemptive antifungal therapy in case of positive results.
